# Moxifloxacin Activates the SOS Response in *Mycobacterium tuberculosis* in a Dose- and Time-Dependent Manner

**DOI:** 10.3390/microorganisms9020255

**Published:** 2021-01-27

**Authors:** Angelo Iacobino, Giovanni Piccaro, Manuela Pardini, Lanfranco Fattorini, Federico Giannoni

**Affiliations:** 1 Dipartimento di Malattie Infettive, Istituto Superiore di Sanità, Viale Regina Elena 299, 00161 Rome, Italy; angelo.iacobino@iss.it (A.I.); manuela.pardini@ifo.gov.it (M.P.); lanfranco.fattorini@iss.it (L.F.); 2 Organismo Notificato Unificato, Istituto Superiore di Sanità, Viale Regina Elena 299, 00161 Rome, Italy; giovanni.piccaro@iss.it

**Keywords:** *Mycobacterium tuberculosis*, SOS response, DNA repair, fluoroquinolone, moxifloxacin

## Abstract

Previous studies on *Escherichia coli* demonstrated that sub-minimum inhibitory concentration (MIC) of fluoroquinolones induced the SOS response, increasing drug tolerance. We characterized the transcriptional response to moxifloxacin in *Mycobacterium tuberculosis*. Reference strain H37Rv was treated with moxifloxacin and gene expression studied by qRT-PCR. Five SOS regulon genes, *recA*, *lexA*, *dnaE2*, *Rv3074* and *Rv3776,* were induced in a dose- and time-dependent manner. A range of moxifloxacin concentrations induced *recA*, with a peak observed at 2 × MIC (0.25 μg/mL) after 16 h. Another seven SOS responses and three DNA repair genes were significantly induced by moxifloxacin. Induction of *recA* by moxifloxacin was higher in log-phase than in early- and stationary-phase cells, and absent in dormant bacilli. Furthermore, in an H37Rv fluoroquinolone-resistant mutant carrying the D94G mutation in the *gyrA* gene, the SOS response was induced at drug concentrations higher than the mutant MIC value. The 2 × MIC of moxifloxacin determined no significant changes in gene expression in a panel of 32 genes, except for up-regulation of the *relK* toxin and of *Rv3290c* and *Rv2517c*, two persistence-related genes. Overall, our data show that activation of the SOS response by moxifloxacin, a likely link to increased mutation rate and persister formation, is time, dose, physiological state and, possibly, MIC dependent.

## 1. Introduction

*Mycobacterium tuberculosis* (Mtb) is a major human pathogen, with 10 million new cases and 1.5 million deaths reported in 2018 [1]. Anti-tuberculosis (TB) therapy consists of a six months treatment with four drugs, unless infection with a drug-resistant strain occurs, or resistance arises during therapy. The insurgence of drug resistance depends on various causes, including poor compliance with medication regimens by the TB patients, a condition that may select genotypically-resistant mutant strains [2]. However, such a long therapy is also related to other factors including the difficulty for the drugs to penetrate inside the cellular and caseous granulomas, where mycobacteria are confined by the host immune response [3,4]. For this reason, inside the granulomas drug concentrations may not be optimal to kill Mtb, in particular since physical and biological conditions such as low oxygen concentration may cause a growth arrest termed dormancy [5,6]. Dormant Mtb is a form of resistance of the microorganism to adverse conditions enabling it to develop phenotypic resistance (drug tolerance) to several drugs [7,8], a condition also termed persistence. Studying the transcriptional response to different drug concentrations may help to identify genes potentially involved in drug persistence or even in the emergence of drug resistance. Studies in *Escherichia coli* showed that a sub-MIC of the fluoroquinolone ciprofloxacin, a drug inhibiting the DNA gyrase, induced the SOS response gene regulon and increased the number of persisters in the population [9,10]. The SOS response, that encompasses a set of genes regulated by the proteins RecA and LexA, plays multiple functions mainly in repair mechanisms in response to DNA damage LexA is a transcriptional repressor that binds to the promoters of the SOS genes, preventing transcription. When DNA damage occurs, RecA binds to single stranded DNA triggering self-cleavage of LexA, which induces transcription of a cascade of SOS genes encoding repair proteins, including *recA* and *lexA*. In *E. coli*, among the induced genes, *tisB* encodes for a toxin associated with increased number of persisters in the population in response to sub-MIC concentrations of ciprofloxacin [10]. Bacterial genomes contain many toxin genes involved in programmed cell death and in cell growth arrest, protecting the cell against multiple stresses and whose expression is controlled by their cognate antitoxin gene [9,11]. In Mtb, 79 potential toxin-antitoxin (TA) systems were identified, suggesting a key role of these genes in survival to adverse conditions [12].

In Mtb, the activation of the SOS response by fluoroquinolones sub-inhibitory concentrations is known [12]. In this study, we characterize the optimal conditions that induce the SOS genes in response to the fluoroquinolone moxifloxacin (MX), an important and widely used second line drug in TB therapy. In particular, we focus on drug dose, time of exposure, physiological state of Mtb cells and on how a MX-resistant mutant responds to the drug.

## 2. Materials and Methods

### 2.1. Bacterial Cultures

*M. tuberculosis* reference strain H37Rv (ATCC 27294) was used for all the experiments. Aerobic actively replicating cultures were obtained by growing the strains in Dubos Tween-albumin (DTA) broth prepared from Dubos broth base and Dubos medium albumin (Difco, Detroit, MI, USA) and collected at different growth conditions. Twelve-day-old and 19-day-old hypoxic (H12 and H19, respectively) non-replicating (dormant) bacilli were generated in DTA broth incubated at 37 °C under stirring conditions (120 rpm) in glass tubes tightly closed with screwed caps and tight rubber caps, as previously described [13,14]. The number of colony forming units (CFUs) in each culture condition was determined by plating 10-fold dilutions on Middlebrook 7H10 (Difco) agar plates and counting colonies after 21 days of incubation at 37 °C in 5% CO_2_. Moxifloxacin was purchased from Sigma-Aldrich (St. Louis, MO, USA). MX was added to aerobic and non-replicating cultures at various concentrations for the time indicated in each experiment, before CFU determination and RNA extraction. The MIC of MX for the strain H37Rv, 0.125 μg/mL, was previously determined [7]. For hypoxic cultures, the drug was added by using a syringe to avoid the air to enter the tubes, as previously described [13]. In all experiments control cultures without drug were set up.

### 2.2. Isolation of Mutant H37Rv gyrA (D94G)

The MIC of an Mtb colony grown on plates containing high MX concentrations, determined as previously described [7], was 2 μg/mL. The clone was isolated, cultured on Middlebrook 7H9 liquid medium (Difco), the DNA extracted, amplified with *gyrA* specific primers as previously described [7] and sequenced. Automated DNA sequencing was performed on a 3730 DNA analyzer with 3730 Data Collection v3 software (Life Technology, Paisley, UK). Sequence alignment showed that the resistant clone carried a single nucleotide mutation in codon 94, from GAC to GGC, which changed the amino acid from aspartate to glycine, conferring fluoroquinolone resistance [7]. The clone, termed H37Rv *gyrA* (D94G), was used in parallel assays with the H37Rv parental strain.

### 2.3. RNA Extraction, Reverse Transcription and qPCR

An aliquot from each culture was centrifuged and bacterial pellets were resuspended in 1 mL TRIzol Reagent (Invitrogen, Carlsbad, CA, USA) and transferred to 1.5 mL screw cap tubes containing approximately 500 μL volume of 0.1 mm zirconia/silica beads (Bio-Spec Products Inc., Bartlesville, OK, USA). *M. tuberculosis* cells were disrupted through 6 cycles of 50 s each at maximum speed, using a Mini-Beadbeater-8 apparatus (BioSpec Products Inc., Bartlesville, OK, USA). Lysates were then added with chloroform and the upper phase was loaded on SV Total RNA Isolation System (Promega, Madison, WI, USA) in line with manufacturer’s instructions. The RNA was eluted in H_2_O and treated directly with 10 U of RQ1 RNase-free DNase (Promega, Madison, WI, USA) at 37 °C for 1.5 h using the supplied buffer before a phenol-chloroform extraction and ethanol precipitation. From 1 to 2 μg total RNA was resuspended in 4 μL of H_2_O, and 0.5 μg random hexamers (Promega, Madison, WI, USA) were added up to a final volume of 5 μL, then RNA was heated at 70 °C for 5 min, chilled on ice and reverse-transcribed in a final volume of 20 μL using 1 μL ImProm-II reverse transcriptase (Promega, Madison, WI, USA) in ImProm-II Reaction Buffer containing 0.5 mM dNTPs and 3 mM MgCl_2_. Quantitative PCR assays from Mtb cDNA were performed in an iCycler iQ (Bio-Rad, Hercules, CA, USA) with iQ SYBR Green Supermix (Bio-Rad, Hercules, CA, USA) and 500 nM of each primer for 40 cycles as follows: 40″ at 95 °C, 40″ at 59 °C, and 50″ at 72 °C for 40 cycles. A final melting curve was performed to analyze the product size and quality of the amplification. First, all cDNAs were normalized to use a cDNA amount corresponding to a Ct value for 16S rRNA between 10 and 11. To determine the relative expression of each Mtb gene examined at different conditions and time points, the ΔCt was calculated by subtracting the 16S rRNA threshold cycle (Ct) from the Ct obtained for each gene in each cDNA sample. Next, the ΔΔCt was calculated by subtracting the ΔCt for each condition to the ΔCt of the control condition. Finally, the fold-change compared to control was calculated using the formula 2^−ΔΔCt^. All reactions were performed in triplicate and all RNAs were tested by qPCR for the absence of genomic DNA contamination using non-reverse-transcribed RNA. Primer pairs were tested for amplification efficiency compared to control 16S, using serial dilutions of H37Rv genomic DNA.

### 2.4. Statistical Analysis

The significance of the differences between fold-changes in the gene expression and CFUs was assessed by the Student’s *t* test *p* values of ≤0.05 were considered significant. A single experiment is always presented, performed in triplicate independent replicates and repeated three times for validation.

## 3. Results

### 3.1. Characterization of SOS Gene Response to Moxifloxacin

**Figure 1:**
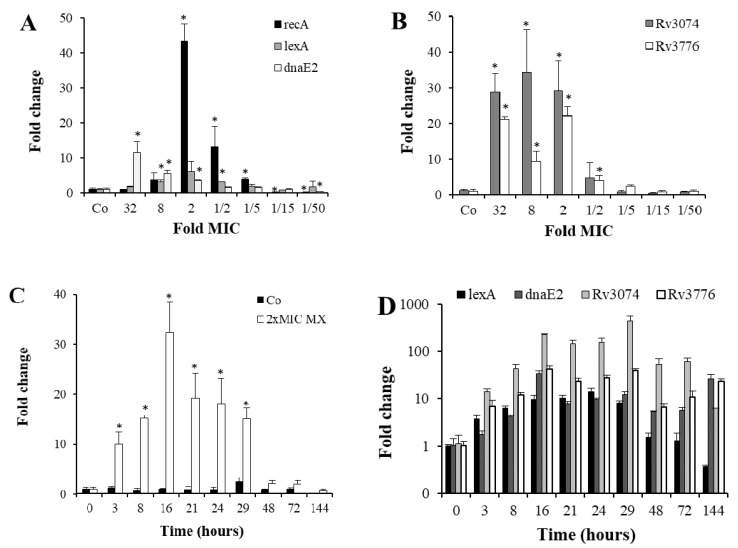

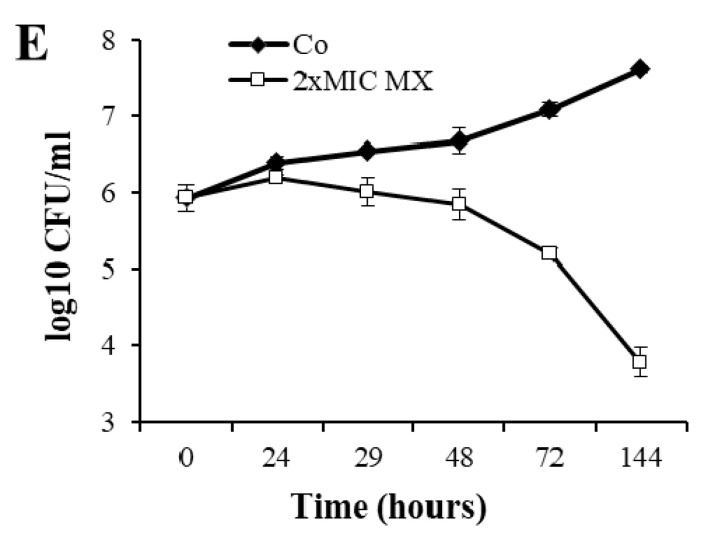
Dose- and time-dependent induction of the SOS response by MX. (**A**) Relative expression of *recA*, *lexA* and *dnaE2* genes compared to untreated control cells, determined by qRT-PCR after overnight incubation of Mtb, with decreasing MX concentrations indicated as fold MIC values. (**B**) Expression of *Rv3074* and *Rv3776* genes in the same experiment as in panel A. (**C**) Time course of *recA* expression by qRT-PCR in Mtb cells incubated with 2 × MIC (0.25 μg/mL) of MX. Gene expression was compared to control cells incubated without MX (Co). (**D**) Gene expression of *lexA*, *dnaE2*, *Rv3074* and *Rv3776* in Mtb cells incubated with 2 × MIC of MX in a time course experiment. All values were significantly different (*p* ≤ 0.02) from time 0 except for *dnaE2* at 3 h and *lexA* at 48 and 72 h. (**E**) CFU counts of untreated and 2 × MIC of MX-treated Mtb cells. A representative experiment of each assay is shown. Error bars show standard deviations, (*) indicates *p* ≤ 0.02 compared to each appropriate control.

In the qRT-PCR assays shown in Figure 1, we demonstrated the activation of five SOS response genes by MX and characterized the optimal parameters for maximal gene induction. In preliminary experiments, we found that overnight incubations with MX up-regulated SOS genes in the MX-susceptible Mtb H37Rv strain (MIC of 0.125 µg/mL) [7]. Based on this observation, we treated mid-log phase Mtb H37Rv cultures with MX concentrations ranging from 32 × MIC (4 μg/mL) to 1/50 × MIC (0.0025 μg/mL), and measured the expression of three SOS response genes: *recA*, *lexA* and *dnaE2*. All three genes were up-regulated in a dose dependent manner (Figure 1A), though following a different pattern of induction, since *recA* and *lexA* peaked at 2 × MIC, whereas *dnaE2* showed a maximal activation at 32 × MIC. Two other genes containing a *lexA* box consensus sequence in their promoters, *Rv3074* and *Rv3776* [3], were strongly up-regulated by MX, with a dose-response pattern different from that of *recA* (Figure 1B), since the highest expression was observed at high MX concentrations, from 2× to 32 × MIC. Interestingly, at very low MX concentrations (1/15 and 1/50 × MIC) *recA* was significantly down-regulated compared to control cells.

Then, using the MX concentration that showed the highest *recA* induction (2 × MIC: 0.25 μg/mL), a time-course analysis was performed to determine the optimal incubation time to obtain the highest *recA* signal. Despite a strong induction was detected as soon as three hours after MX addition, the peak was observed after 16 h (Figure 1C) and high levels of induction lasted up to 29 h of drug exposure. After 48 h, when CFUs started to decrease more dramatically (Figure 1E), *recA* mRNA levels were back to control cells. *RecA* mRNA levels were constant in control cells, decreasing only at 144 h, approaching stationary phase. Also *lexA* induction was abolished after 48 h of incubation, whereas *dnaE2*, *Rv3074* and *Rv3776* peaked between 16 and 29 h, but displayed higher mRNA levels up to 144 h of incubation with 2 × MIC of MX (Figure 1D).

We also examined eight more genes that had been previously described as belonging to the SOS response regulon [3]. Seven of them were significantly upregulated, in particular a strong induction was observed for *Rv1378c*, *Rv2719c* and *Rv3395c* (Table 1). Two of the genes analyzed, *Rv0336* and *Rv0515*, were undistinguishable, due to a single nucleotide difference in their sequence. In addition, the analysis of five DNA repair genes showed the induction by MX in three of them, *ssb*, *ruvC* and *radA*.

### 3.2. Mtb Response to Moxifloxacin at Different Growth Phases

**Figure 2:**
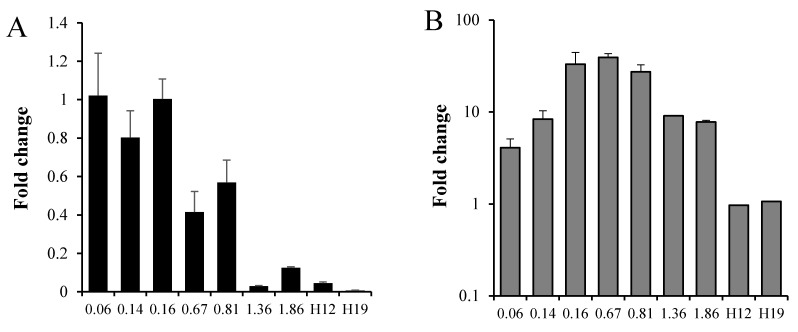
Role of growth phase on *recA* gene expression and induction by MX. A fresh culture of Mtb H37Rv was set up from early phase to stationary phase. At each optical density (OD) value indicated in the X-axis, culture aliquots were incubated with or without 2 × MIC (0.25 μg/mL) of MX for 16 h. In parallel, hypoxic day 12 (H12) and day 19 (H19) Wayne dormancy cultures were treated with 2 × MIC of MX for 16 h in constant hypoxic conditions. At the end of each incubation period, total RNA was extracted and gene expression determined by qRT-PCR. Early log-phase cells: OD 0.06 and 0.14. Log-phase cells: OD from 0.16 to 0.81. Stationary phase cells: OD from 1.36 to 1.86. (**A**) Fold-change in recA gene expression compared to early log phase cells (OD of 0.06). (**B**) *RecA* fold-changes in Mtb H37Rv incubated for 16 h with 2 × MIC of MX compared to the corresponding growth phase untreated cells. An equal statistically significant difference between *recA* expression of MX-treated and untreated cells was observed for all aerobic conditions (*p* ≤ 0.01) except for OD 1.86 (*p* ≤ 0.02).

Next, we examined the *recA* induction by MX at different growth phases of Mtb (Figure 2). Aerobic cultures and hypoxic 12- and 19-day-old cultures (H12 and H19, respectively), prepared according to the Wayne dormancy model, were set up. In the aerobic growth curve, *recA* basal expression slightly decreased with time, with very low levels in the stationary phase, at OD of 1.36 and 1.86, corresponding to seven and nine days of culture. Moreover, *recA* basal mRNA levels were also low in H12 and H19 non-replicating hypoxic (dormant) cells (Figure 2A). When, at each growth phase, 2 × MIC of MX was added to the culture and incubated for an additional 16 h and *recA* mRNA levels compared to untreated Mtb at the same growth phase, the responsiveness of Mtb to MX changed, being the highest in mid-log phase bacteria (ODs of 0.16, 0.67 and 0.81), showing a *recA* increase of 33-, 39- and 27-fold, respectively, compared to untreated Mtb (Figure 2B). At early (OD 0.06 and 0.14) and stationary (OD 1.36 and 1.86) phases MX had a more moderate effect on *recA* induction. Finally, hypoxic cultures showed no induction after treatment with the drug.

### 3.3. SOS Response in a Moxifloxacin Resistant Strain

**Figure 3:**
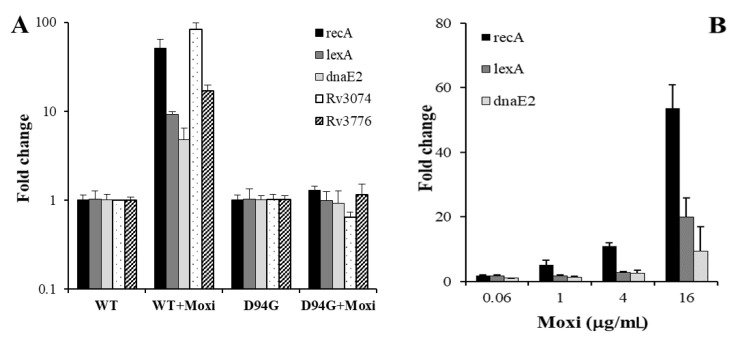
SOS response by MX in the H37Rv *gyrA* (D94G) mutant strain. (**A**) Expression of the SOS response genes determined by qRT-PCR from mid-log phase wild-type (WT) and mutant (D94G) H37Rv strains after treatment with 0.25 μg/mL of MX for 16 h. Values of WT + MX and D94G + MX were normalized to those of each corresponding control, WT and D94G, respectively. (**B**) Changes in gene expression of *recA*, *lexA* and *dnaE2* after incubation of the H37Rv D94G strain with variable concentrations of MX (0.06, 1, 4, 16 μg/mL) for 16 h.

The information that *recA* was induced by MX in the MX-susceptible H37Rv strain prompted us to measure *recA* induction by MX also in a MX-resistant H37Rv strain. To this end, we isolated on an antibiotic containing plate the strain H37Rv *gyrA* (D94G) harboring a single nucleotide mutation in the *gyrA* gene, which is known to confer fluoroquinolone resistance [7]. Interestingly, 16 h of incubation with 0.25 μg/mL, corresponding to 2 × MIC of MX in wild-type H37Rv, did not induce the SOS response in this mutant strain (Figure 3A). Still, the possibility that higher MX concentrations, close to the MIC of the mutant strain, activated the SOS response could not be ruled out. To this purpose, we incubated H37Rv D94G strain with variable doses of the drug. As shown in Figure 3B, higher MX concentrations strongly activated the SOS response, suggesting a close link between MIC values and the drug effect on transcription.

In *E. coli*, fluoroquinolone-resistant strains showed a constitutive higher expression of the SOS response genes [15]. We also observed in the mutant strain a mild induction in the basal expression of the SOS regulon when compared it to its parental strain, in particular for *recA* and *dnaE2*, which showed a 3.8- and a 3.7-fold increase, respectively (data not shown in Figure 3). No changes were observed for *lexA*.

### 3.4. General Transcriptional Response to Moxifloxacin

Finally, to further characterize the transcriptome associated with the SOS response, we examined by qRT-PCR the expression of a panel of 31 genes involved in different cellular functions such as transcription, early genes, dormancy, replication, porins, pumps, toxins and persistence (Table 2), to ascertain the physiological conditions of MX-treated Mtb and the induction of some genes potentially involved in persistence formation. We observed that the expression of most genes was constant or only slightly down-regulated by exposure to 2 × MIC of MX. However, some genes were mildly but significantly up-regulated by the drug, including the toxin *relK* (5.86 times) and the persistence related genes *Rv3290c* and *Rv2517c* (3.83 and 2.64 times, respectively). The transcription associated gene *phoP* was upregulated by 3.29 times, but the level was not statistically significant.

## 4. Discussion

In this study, we investigated the transcriptional response of Mtb to MX. All genes examined containing a LexA box and belonging to the SOS regulon were activated at close-to-MIC levels of MX, with the exception of *whiB2*, confirming the doubts that the promoter of this gene is bound by LexA [3]. Interestingly, not all genes studied belonging to the regulon were activated at the highest level at the same MX concentration. In fact, whereas *recA* and *lexA* were optimally activated at 2 × MIC (0.25 μg/mL), a shift to higher concentrations (up to 32 × MIC, 4 μg/mL) for *dnaE2*, *Rv3074* and *Rv3776* was observed. The maximum drug concentration in serum (C_max_) of MX is around 4 μg/mL [16]. Whether or not this response is still *recA*-dependent needs to be ascertained. Moreover, in a time course experiment we demonstrated that *recA* induction was rapid and sustained for about 1 day, until killing by the drug occurred.

The highest *recA* induction by MX was observed in mid-log phase bacilli, with progressively decreasing gene induction in stationary phase cells. Instead, no *recA* activation in nonreplicating, dormant stages was detected, as expected from the knowledge that fluoroquinolones are only moderately active against dormant Mtb [13]. These observations lead to the conclusion that the activation of the SOS response correlate to the rate of Mtb replication, when GyrA is at its maximal activity. Moreover, since it was shown that the mutation D94G causes a decrease in drug-gyrA affinity [17], our assay demonstrated that the activation of the SOS regulon was dependent on direct drug-target interaction, since *recA* induction was totally abolished in the H37Rv *gyrA* (D94G) mutant at MX concentrations close to the MIC of the parental strain. Indeed, when we used drug concentrations close to the MIC of the mutant strain, a strong SOS response induction was observed. Despite the presence of additional mutations affecting the regulation of the SOS response being unable to be ruled out, this observation suggests that each Mtb strain should have an optimal drug concentration, depending on the MIC of the strain that induces the SOS response.

The induction of *dnaE2* could be envisaged as a “bridge” between phenotypic and genotypic resistance. Indeed, this gene encodes for an error-prone DNA polymerase and its activation is linked to increased mutation rate, a way to generate drug resistant strains at high frequency in response to a drug [2]. Induction of *dnaE2* was found in Mtb persisters also by other investigators [16]. Moreover, despite *Rv3074* and *Rv3776* functions still being unknown, they both share homology to the superfamily of endonucleases containing histidine asparagine motifs (HNH); in other organisms some of these endonucleases cleave DNA only when RecA protein is bound [18]. High frequency of generation of resistant mutants in Mtb persistent cells was recently demonstrated for rifampin and MX [19]. For the first drug, the mechanism was shown to be linked, at least in part, to reactive oxygen species formation. For MX, the mechanism could be at least in part SOS response dependent.

Mtb responds to DNA breaks induced by DNA damaging agents by overexpressing DNA repair genes. We showed that some of these genes respond to MX treatment, in particular *ssb*, *ruvC* and *radA*. Ssb binds to single-strand DNA, interacting withrecA in DNA repair by homologous recombination [20], but its promoter does not have a clear lexA binding site, suggesting that additional transcriptional mechanisms, recA independent, take place in the presence of different DNA damaging agents [21].

In *E. coli*, it was demonstrated that the toxin tisB, an antimicrobial peptide that apparently forms an ion channel in the cell membrane, is over-expressed after the SOS response induction by fluoroquinolonesand plays a key role in persister formation [5]. However, *tisB* Protein BLAST versus H37Rv genome did not find any obvious similar protein. In Mtb, we failed to observe a consistent increase in persister numbers after low-dose MX treatment; however, since the induction of persistence was recently observed after silencing Mtb *gyrA* and *gyrB* [22], additional experiments should test this hypothesis.

Since low-dose drug treatment is associated to slow growth, we tested the effect of MX on transcription factors, log-phase and dormancy genes, and on many genes potentially involved in persistence mechanisms. Overall, during incubations with 2 × MIC of MX, most genes remained constant, including those activated in the dormancy state, suggesting that the activation of the SOS response is a highly specific event. We detected the up-regulation of *relK*, an Mtb toxin, and of two out of the five genes identified as commonly up-regulated in persistence and different dormancy models, namely *Rv2517c*, encoding a hypothetical protein, and *Rv3290c* (*lat*), encoding an L-lysine-epsilon-aminotransferase [10]. Further studies need to establish the importance of these genes in response to DNA damage.

Overall, our data show that close-to-MIC concentrations of the DNA-damaging agent MX induced a strong SOS response, potentially driving an increased mutation rate and persister formation.

## Figures and Tables

**Table 1:** mRNA fold-changes (FC) and standard deviations (SDs) determined by qRT-PCR for SOS response and DNA repair genes in aerobic Mtb H37Rv cells treated with 2 × MIC (0.25 μg/mL) of MX for 16 h, compared to control cells grown in the absence of the drug F and R: forward and reverse primers, respectively (*), *p* < 0.02; (**), *p* < 0.002; (-), not significant.

Category	Rv No.	Gene	FC ± SD	*p*	F	R
SOS response	*Rv1378c*	*-*	43.85 ± 8.93	**	ACGCCCACCGGGATGTACTA	GAGGGCGACACCGATTCTGG
	*Rv0336*	*-*	6.25 ± 0.93	**	TGATCACCGCCGAACTGGTG	CCAGCGACACGTCAGATCCC
	*Rv0515*	*-*	6.25 ± 0.93	**	TGATCACCGCCGAACTGGTG	CCAGCGACACGTCAGATCCC
	*Rv1000c*	*-*	8.09 ± 1.41	**	ACATCTACGGCGGCGAACTG	CCGTCGCGGTAGTAGCACAG
	*Rv2719c*	*-*	40.58 ± 5.61	**	CCGCGGCGATTACTCTCTGG	GGACCGCCACGTCATACAGG
	*Rv3395c*	*-*	70.31 ± 4.87	**	GGACGGTGGGAGTGCTGTC	ACCGATGCCACCATGCTCAG
	*Rv0427c*	*xthA*	4.49 ± 0.18	**	ATCGCACTGATGGGCGACTG	AATTGCGCGTCGACAATGGC
	*Rv3260c*	*whiB2*	0.32 ± 0.02	**	GGAAGCCACCGACCAATG	CCAGGGCGTACTCCAGAC
DNA repair	*Rv0054*	*ssb*	15.58 ± 3.70	*	CGTCAGACCGGCGAATGGAA	TCGATGACGGTGCGCTTCTC
	*Rv1638*	*uvrA*	2.55 ± 1.50	-	CCCGAGATTCTGGCGGTGAC	GCGACCGGATCTCCTTGAGC
	*Rv2594c*	*ruvC*	9.90 ± 1.23	**	ACGTGGTGTCGACGTGCATT	GACCATCGCGGTGACCTGAG
	*Rv3585*	*radA*	12.18 ± 1.41	**	CGGTATCGTTCCCGGTTCGG	CTGACCGGCGGATTCCTCAC
	*Rv2821c*	-	0.85 ± 0.21	-	AGCAGGCTGCCGATGATTCC	GAGCTTCGTGTCGCGGAAGA

**Table 2:** mRNA fold-changes (FC) and standard deviations (SDs) determined by qRT-PCR for each indicated gene in aerobic Mtb H37Rv cells treated with 2 × MIC (0.25 μg/mL) of MX for 16 h, compared to control cells grown in the absence of the drug F and R: forward and reverse primers, respectively (*), *p* < 0.02; (**), *p* < 0.002; (-), not significant. The SOS response genes refer to data shown in Figure 1.

Function	Rv No.	Gene	FC ± SD	*p*	F	R
**SOS Response**	*Rv2737c*	*recA*	43.41 ± 5.02	**	GCGGTGGAATGAAGCAGGTC	GTTGGCAGCACTTCTCGGATC
	*Rv2720*	*lexA*	6.09 ± 2.94	**	GCCGAGGAAGCCGTTGAAG	CGATCACCTTGAGCAGGAACAG
	*Rv3370c*	*dnaE2*	3.59 ± 0.17	**	GTTCTACTCGGCGTGGTTCAAG	CAGCGACTGCGGCGAATAG
	*Rv3074*	*-*	29.28 ± 8.38	**	CATTAGATGCCGAGTTATGTGG	GCCGAATGGTGACCGTAC
	*Rv3776*	*-*	22.19 ± 2.38	**	CTGCGGCTGCGGTAATTC	CGGATAGGCGTGGGTCAG
**Transcription**	*Rv0667*	*rpoB*	0.91 ± 0.32	-	TCGCCGACCTGGATGAGC	CGAAGTGTCGCGCACCTC
	*Rv2703*	*sigA*	0.89 ± 0.26	-	AGTCGGAGGCCCTGCGTCAA	GCCAGCCTCGATCCGCTTGG
	*Rv2710*	*sigB*	0.83 ± 0.22	-	ATCCGCCAGGCCATCACC	TCCTCATCGGTGGCTTCGC
	*Rv1221*	*sigE*	0.79 ± 0.19	-	GCCCAACCCCGAGCAGATC	CGTACCGTCCCGAGCTTCAC
	*Rv3286c*	*sigF*	0.86 ± 0.12	-	GTTCCTACCACACCTTGTCCATC	CCCAGGGTGTCTGTGATTGC
	*Rv0757*	*phoP*	3.29 ± 1.00	-	CGGGATGGACGGCTTTGG	ATCTTGTCCTGTAGCGAGTCAC
**Early Genes**	*Rv3875*	*esat-6*	0.68 ± 0.1	-	CGCAATCCAGGGAAATGTCACGTC	GTACGCCTCCGAACCGCTACC
	*Rv1886c*	*fbpB*	0.74 ± 0.08	*	CTGTAGTCCTTCCGGGCCTGGT	CACCGCTCTGGAACTGAACCTTGA
**Dormancy**	*Rv2031c*	*acr*	1.47 ± 0.32	-	CCGAGCGCACCGAGCAGAAG	GCCTTAATGTCGTCCTCGTCAGCA
	*Rv1738c*	*-*	0.56 ± 0.16	-	TGCGGCGACCAGTCGGATCA	GCCAGGCCAACACCCACCAATT
**Replication**	*Rv0006*	*gyrA*	1.07 ± 0.63	-	TCGCCCAGGTCATCCAGATT	CAGCAGCAGGTCGTCGC
**Porins**	*Rv0431*	*-*	0.64 ± 0.37	-	TTCTCGGCGTCGTCTTCC	TTCTGTGCCTGAGATGTTGTAG
	*Rv1352*	*-*	1.1 ± 0.13	-	CGATCACGCTCGCACCTG	GGACACCCTGCCGAACAC
	*Rv1698*	*-*	0.35 ± 0.18	-	CGGCAAGTCGGTGGTCATC	CTCGGCGGAGTTGGCTTC
**Pumps**	*Rv1217c*	*-*	0.52 ± 0.16	-	GGTTGTTATCGGCGGTGAC	CCAGGTTGAGCAGCATCTG
	*Rv1410c*	*-*	0.64 ± 0.11	-	GTTCATCATCGGCTCGGTAGTG	CCAGCGTGATCGGCAATAGC
	*Rv2846c*	*efpA*	1.15 ± 0.31	-	CTTGACCACGCCTACACCTAC	GATCGCTTCCTTGACCTCCTG
	*Rv3065*	*mmr*	1.78 ± 0.18	-	AAGCACGGAAGGGTTCACTC	TACGGCGACCAGCACAATG
**Toxins**	*Rv2583c*	*relA*	0.45 ± 0.18	*	GCAGCAGTTCGTGGTGTCG	CTCGCGGGCCATCGCATC
	*Rv1246c*	*relE*	0.71 ± 0.49	-	GAGCGACGACCATCCCTACC	CGGCTTGCCCAACCTATGC
	*Rv2866*	*relG*	0.66 ± 0.17	-	CCGTGCGGTTCACCACAAC	TACAGCAGGCGGTACGTTCC
	*Rv3358*	*relK*	5.86 ± 1.10	**	AGGTGAGTTGTCGGGATACTGG	GCCTTCAGCATCGTGACTTCG
	*Rv1102c*	*mazF3*	0.68 ± 0.07	-	GAATCAACCGTCAGTCGTCAGC	GCGAGCAGGTAGCCGATTTG
	*Rv1495*	*mazF4*	0.49 ± 0.02	-	GTTGCGTGGTCAGGTCTATCG	GGTTGCGGGCGTTGTTGG
	*Rv1991c*	*mazF6*	1.13 ± 0.20	-	GGTGCTCGTAATCCAGTCAGATC	GTCGGTGAGGTCAGTCTTGTTG
	*Rv2801c*	*mazF9*	0.66 ± 0.32	-	CGAGGTAGCGAAGCGAACAAC	CGGATAGACCTTGGCGATGTTG
**Persistence**	*Rv0251c*	*acr2*	1 ± 0.55	-	CGACGAGCACACGCAAGACG	CGGAACGAGCGGCGGAATG
	*Rv2517c*	*-*	2.64 ± 0.18	**	GCTACACCCGCTTCTACAACC	CCGCCGTTCCTTCTTGCTC
	*Rv1152*	*-*	0.37 ± 0.06	*	GGACGCTTCGGCACTTTC	CGCATCGCATCGGACTTC
	*Rv2497c*	*pdhA*	0.69 ± 0.45	-	ACGCCTGGACGAGGACTC	ACGCACGGTGTGGTGAAC
	*Rv3290c*	*lat*	3.83 ± 0.41	**	CGTGAAGTCCGTCGCTCTTG	CCGAGGAGGCAACGAATGTG

## Data Availability

Not applicable.
